# Application of the Gaussian Process Regression Method Based on a Combined Kernel Function in Engine Performance Prediction

**DOI:** 10.1021/acsomega.2c05952

**Published:** 2022-11-03

**Authors:** Xiuyong Shi, Degang Jiang, Weiwei Qian, Yunfang Liang

**Affiliations:** † School of Automotive Studies, Tongji University, Shanghai201804, China; ‡ China Ship Scientific Research Center, Wuxi214082, China

## Abstract

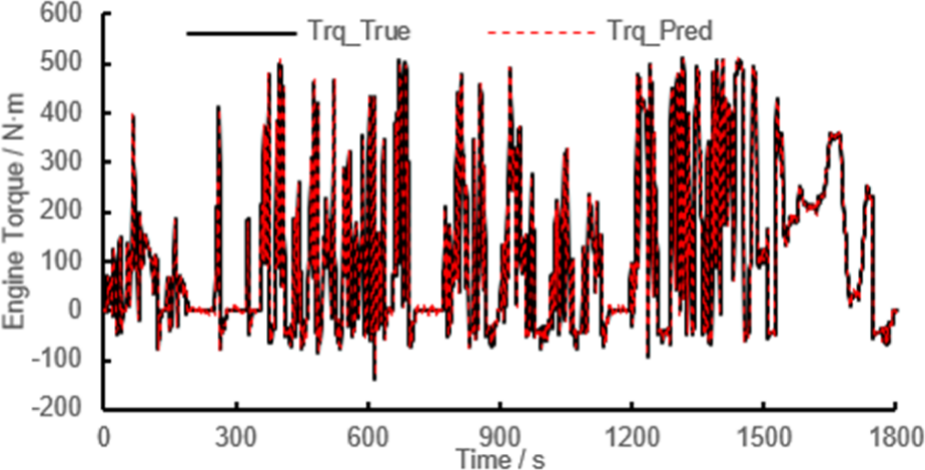

At present, regression modeling methods fail to achieve higher simulation accuracy, which limits the application of simulation technology in more fields such as virtual calibration and hardware-in-the-loop real-time simulation in automotive industry. After fully considering the abruptness and complexity of engine predictions, a Gaussian process regression modeling method based on a combined kernel function is proposed and verified in this study for engine torque, emission, and temperature predictions. The comparison results with linear regression, decision tree, support vector machine (abbreviated as SVM), neural network, and other Gaussian regression methods show that the Gaussian regression method based on the combined kernel function proposed in this study can achieve higher prediction accuracy. Fitting results show that the *R*^2^ value of engine torque and exhaust gas temperature after the engine turbo (abbreviated as T4) prediction model reaches 1.00, and the *R*^2^ value of the nitrogen oxide (abbreviated as NOx) prediction model reaches 0.9999. The model generalization ability verification test results show that for a totally new world harmonized transient cycle data, the *R*^2^ value of engine torque prediction is 0.9993, the *R*^2^ value of exhaust gas temperature is 0.995, and the *R*^2^ value of NOx emission prediction result is 0.9962. The results of model generalization ability verification show that the model can achieve high prediction accuracy for performance prediction, temperature prediction, and emission prediction under steady-state and transient operating conditions.

## 1 Introduction

At present, simulation technology is showing its ability in the automotive field, and the technology has been developed from performance simulation to the application of full-life-cycle simulation of products. Simulation technology has the characteristics of visibility, verifiability, perception, and so forth.^1^ It can be used to accelerate the automotive product development phase and improve system reliability;^2,3^ however, the problem of low simulation accuracy limits its wide application in fields of virtual calibration and hardware-in-the-loop real-time simulation. The main reasons for the low accuracy of engine performance simulation are as follows:

1. Engine performance could abruptly change. Taking carbon monoxide emission as an example, when the exhaust gas temperature and air–fuel ratio exceed a certain limitation, carbon monoxide emission would possibly change abruptly, which brings challenges to traditional Gaussian process regression (GPR) algorithms, support vector machine (SVM), and their covariance functions to reflect the correlation between variables;
2. Engine system is a complex system involving multiple disciplines such as mechanics, thermodynamics, chemistry, and so forth, which brings challenges to the feature extraction of the regression modeling process.

In the field of engine performance prediction, scholars have conducted long-term research. Engine modeling technology can be divided into mechanism modeling technology and regression modeling technology.

Mechanism modeling is a modeling technology based on the physical properties of each component. This technology analyzes the working process of the object and widely adopts the ideal state equation, look-up table, and other methods to establish the airflow process model and thermodynamic process model of the engine.^4−6^

The advantage of the mechanism modeling method is that it helps to understand the characteristics of engine components, the interaction between components, and the effect of components on the engine’s overall performance.^7^ Also, the mechanism modeling method has the following disadvantages:

1. The operation process of the engine is complex, involving multiple disciplines such as mechanics, thermodynamics, chemistry, electronic control technology, and so forth, and the current research fails to clearly understand the combustion process of the engine, which brings great challenges to the mechanism modeling process;
2. The model shows low accuracy, and the calibration process is challenging. Mechanism modeling methods widely adopt approximation or idealization methods such as the ideal state equation and look-up tables for modeling, and many parameters could be obtained only through the data fitting method instead of direct experiments. This makes the model calibration process difficult and the model accuracy low.

Regression modeling is a mathematical modeling method applying statistical methods to quantitatively show the working process.^8,9^ Higher prediction accuracy could be achieved with neural networks, decision trees, SVM, and so forth. Kang and Zhou^10^ studied the relationship between the engine torque and cylinder pressure through the linear regression fitting method and obtained the correlation between the engine torque and cylinder pressure: *P* = 0.0229*N* + 0.9969. Zhang et al.^11^ built a diesel engine emission prediction model with a three-layer BP neural network, and the result showed that the error between the model prediction result and experimental result was less than 9%. Hui and Li^12^ used weighted least-squares method to establish a linear regression model for engine torque prediction. Test results showed that the model prediction error was 7.60%. Li et al.^13^ built an RGF model for engine torque and fuel consumption rate prediction, and the results showed that the prediction error of engine torque under steady-state and transient conditions would be within 5%. Shahpouri et al.^14^ built an engine soot emission prediction model with the regression tree (RT), ensemble of RTs, SVMs, GPR, artificial neural network, and Bayesian neural network, and results showed that the fitting *R*^2^ value of the engine black-box model using GPR and feature selection by LASSO reached 0.96, and the fitting *R*^2^ value of the gray-box model using SVM reached 0.97.

The above-mentioned algorithms have wide applications in the field of machine learning, and many scholars have conducted in-depth research on them. However, the application performance in the field of engine performance prediction needs to be further improved for higher simulation accuracy.

In recent years, GPR has been widely used in the field of nonlinear system modeling. In a Gaussian process, each point in a continuous input space is associated with a normally distributed random variable. A Gaussian process is a random process in which observations appear in a continuous domain.

The kernel function in Gaussian regression characterizes the correlation between variables. As part of the model assumptions, different kernel functions can achieve different fitting results. Commonly used kernel functions include the radial basis function kernel (abbreviated as RBF kernel), Matern kernel, exponential function kernel (exponential kernel), rational quadratic kernel (abbreviated as RQ kernel), periodic kernel, polynomial kernel, and so forth.

Without limiting the form of the kernel function, Gaussian regression is theoretically a universal approximator of any continuous function in a compact space. In addition, Gaussian regression can provide the posterior of the prediction result, and this posterior has an analytical form, so Gaussian regression is a general and analytic model.^15^ Based on the above advantages, people can use the Gaussian regression technology to quickly and efficiently create models of engines, power systems, or any other systems, and people can more conveniently adjust and optimize calibration parameters, reduce the need for calibration development work on the engine test bench or vehicle, so this technology makes powertrain system development more efficient.

Although Gaussian regression has the advantages of generality and analyzability,^16−19^ Gaussian regression is not flexible enough when the data in different areas changes abruptly, and a single kernel function cannot fit effectively.

Based on the above analysis, this study proposes and demonstrates the technical feasibility of the GPR algorithm based on a combined kernel function (Section 2), and a black-box model of a 3.0 L diesel engine is established (Section 3). The engine torque, emissions, and temperature performance are predicted using the method proposed in this study (Sections 4.1 and 4.3), and the prediction accuracy of engine torque by linear regression, decision tree, SVM, neural network, GPR, and the method proposed in this study is compared using the same training dataset in Section 4.2. The generalization ability of the model is validated under transient running conditions, which is not included in the training dataset.

## 2 GPR Technology Based on a Combined Kernel Function

GPR is a major data fitting method in the field of machine learning. Theoretically, this method can provide nonlinear models for any system. Although the model space is infinitely dimensional, the problem of overfitting can be prevented by empirical Bayesian methods, which provide a maximum-likelihood model given a limited set of measurement data. The model fitted by the GPR method is given as a Gaussian probability distribution for each array of input variables. From the weight-space point of view, GPR can be derived from the principle of Bayesian linear regression, that is, for a given set of *N* independent learning samples: ; . Bayesian linear regression is a multiple linear regression model^20^ that satisfies eq 1.
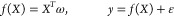
1where ω is the weight coefficient and ε is the residual or noise.

Bayesian linear regression is a linear parametric model, as shown in eq 2, that characterizes the nonlinear relationship between variables; a given function can be used to map *X* to a high-dimensional space.
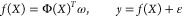
2where ωis the weight coefficient and εis the residual or noise.

Since the mapping space Φ(*X*) has nothing to do with the model weight, it can be directly brought into the result of Bayesian linear regression as shown in eqs 3 and 4.
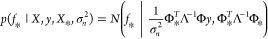
3
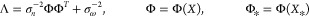
4where  is a likelihood of Bayesian linear regression;  is the normal distribution with a mean value ; and σ denotes the standard deviation.

Using the kernel method, that is, defining the kernel function , eq 3 can be rewritten as eq 5, that is, using GPR to predict the mean and covariance values.
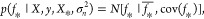

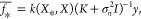
5
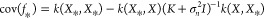


The applicability of a Gaussian process is limited by its basic mathematical assumptions, namely:

1 The dataset obeys a Gaussian distribution;
2 The sample noise is homoscedastic Gaussian noise;
3 Suitable for smooth function fitting;
4 The covariance function is satisfied between different variables of the dataset.

However, the above assumptions are not always met in many application scenarios. For example, when the exhaust gas temperature exceeds a limit, the emission changes abruptly, and the sample noise no longer meets the assumption of homoscedastic noise. For the prediction of mutation signals, the traditional GPR is not flexible enough, and it is difficult for a single kernel function to achieve a higher fitting accuracy. This study takes engine torque prediction based on main injection quantity as an example and analyzes the fitting effect of square exponential kernel function and rational quadratic kernel function, and verifies the technical feasibility of the GPR technique based on the combined kernel function in the application of engine performance prediction.

### 2.1 Squared Exponential Kernel Function

The squared exponential kernel, also called Gaussian kernel or RBF kernel, is the function space expression of the RBF regression model with infinitely many basis functions. The squared exponential kernel function, whose expression is shown in eq 6, is widely applied in GPR and SVM
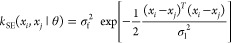
6where σ_l_ is the scale of the signal feature length, which is used to describe the smoothness of the function. When σ_l_ is small, the dynamic response performance of the fitting function is better, but it is accompanied by the risk of overshooting; when σ_l_ is large, the resultant function tends to be smooth.

σ_f_ is the standard deviation of the signal, which is used to characterize the deviation of the fitting function from the signal mean value. When σ_f_^2^ is small, the fitting function deviates from the signal mean value slightly. Whenσ_f_^2^ is large, the fluctuation of the fitting function will become larger.^21^

 can be regarded as the squared Euclidean distance between two eigenvectors; as the value of the squared exponential kernel function decreases with the decrease of distance, its value is limited between 0 and 1 (when *x*_*i*_ = *x*_*j*_, its value would be 1), so it is a ready-made similarity measure. The feature space of a kernel has an infinite number of dimensions.

It can be seen from eq 6 that the squared exponential kernel function is infinitely differentiable, which means that the GPR with the squared exponential kernel function as a covariance function has the mean-squared derivative of all orders; meanwhile, the squared exponential kernel function replaces the inner product of the basis function with a kernel, and the advantage of this function is that the error is relatively controllable when dealing with large datasets with high dimensions. Therefore, the squared exponential kernel function is widely suitable for the modeling of smooth and continuous datasets, but it performs poorly when there are many training samples or when the samples contain many features.^22,23^

### 2.2 Rational Quadratic Kernel

The expression of rational quadratic kernel is shown in eq 7.
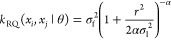
7where σ_l_ is the scale of the signal feature length, α is a positive-valued scale-mixture parameter (α is a positive-valued scale-mixture parameter), and *r* is the Euclidean distance between *x*_*i*_ and *x*_*j*_, which is defined in eq 8.
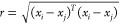
8

The rational quadratic kernel is a linear superposition of infinite square exponential kernel functions. When α → ∞, the rational quadratic kernel is equivalent to the square exponential kernel function with *l* as the characteristic scale. The rational quadratic kernel has a wide scope, which could help to reduce the sensitivity of the model to smaller datasets and improve the generalization ability and dynamic response performance.^24^

### 2.3 Combined Kernel Function

Based on the above analysis, as shown in eq 9, this study intends to construct a new kernel function based on square exponential kernel and rational quadratic kernel, which not only takes advantage of square exponential kernel function for modeling with high-dimensional datasets but also the dynamic response performance of fitting results could be improved by the rational quadratic kernel function.
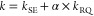
9where α is the weighted coefficient of the rational quadratic kernel function in the combined kernel function.

**Figure 1:**
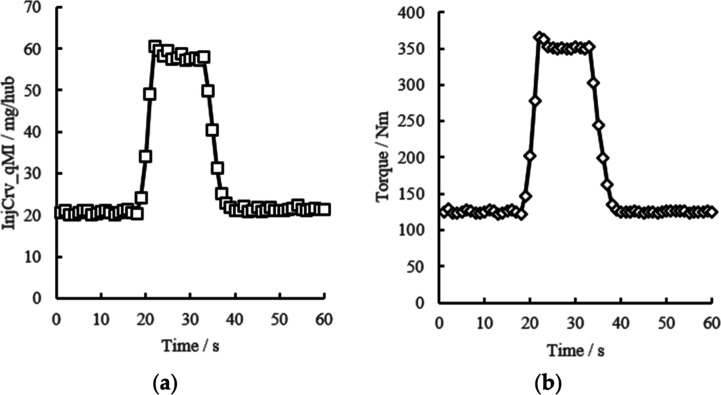
Dataset overview. (a) Main injection quantity; (b) engine torque.

Based on the above analysis, to further verify the fitting performance of the square exponential kernel function, the rational quadratic kernel function, and the combined kernel function, this paper selects the test data of a 3.0 L diesel engine under transient working conditions for verification. There are 60 sample points in total; each point contains two variables: engine main injection quantity and engine torque. The basic information of the engine is shown in Table 1, and the dataset information is shown in Figure 1. It can be seen from Figure 1 that the dataset contains both a relatively smooth stable operation stage and a signal mutation process.

**Table 1:** Engine Basic Information

parameter	value
displacement (L)	2.977
air intake system	turbocharged
cylinder arrangement	in-line
number of cylinders	4
rated power/speed(kW/rpm)	125/2800
compression ratio	16.0:1
fuel injection system	common rail
idle speed (rpm)	800 ± 30
fuel injection pressure (MPa)	200

With the same dataset, different kernel functions are used for engine torque prediction. As shown in eqs 10–13, the root-mean-square error (RMSE), *R*^2^ (goodness of fit), mean square error (MSE), and mean absolute error (MAE) of engine torque deviation value is calculated by comparing the predicted value and the true value to evaluate the fitting performance of different kernel functions.
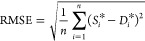
10
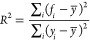
11
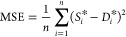
12
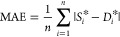
13

**Figure 2:**
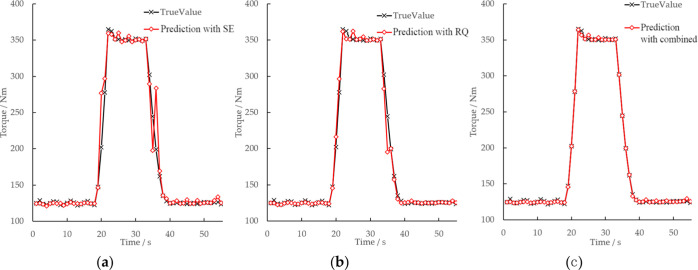
GPR training result. (a) GPR training result with the squared exponential kernel; (b) GPR training result with the rational quadratic kernel; (c) GPR training result with the combined kernel.

**Figure 3:**
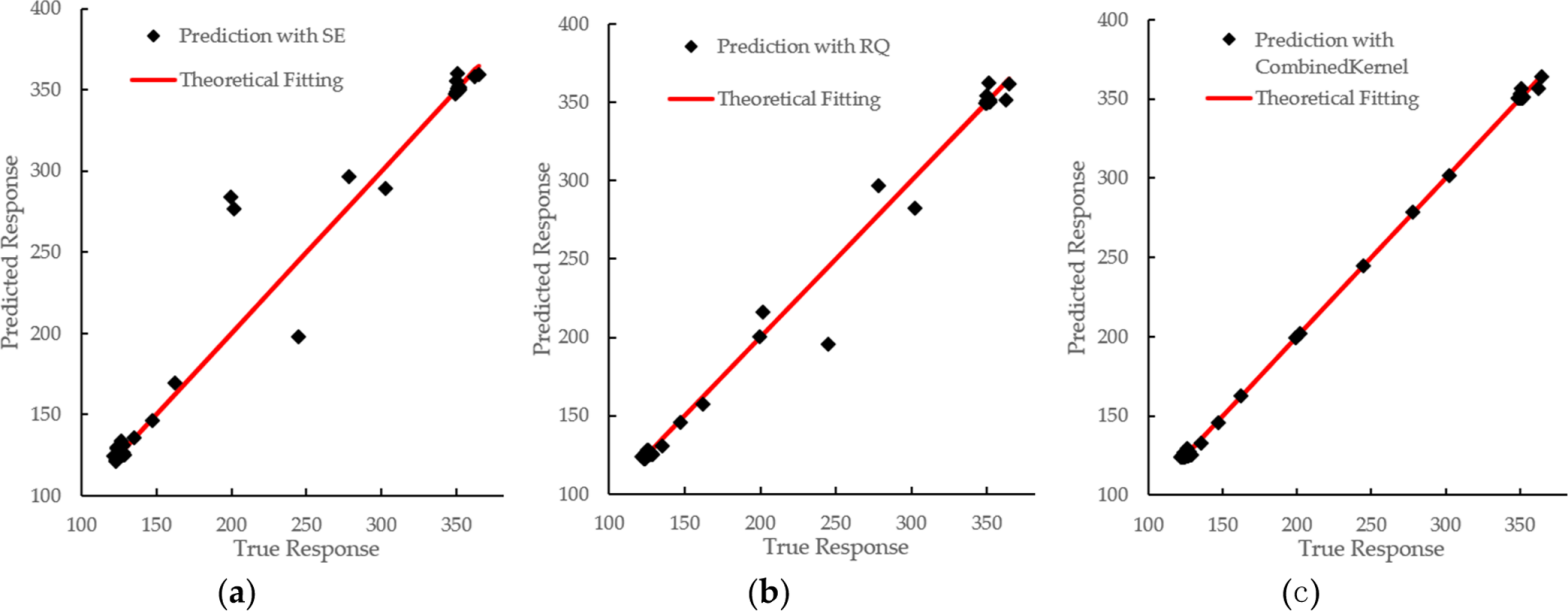
Comparison of predicted results with actual results. (a) GPR training result with the squared exponential kernel; (b) GPR training result with the rational quadratic kernel; (c) GPR training result with the combined kernel.

A reserved crossover method is used in the model training process; the training results of the GPR models using the square exponential kernel function, rational quadratic kernel function, and combined kernel function are shown in Figure 2 and Table 2. As shown in Figure 3, the comparison chart between predicted results and the true value is used in this paper to illustrate the fitting performance of the model at different sample points. The predicted results of the model should theoretically be close enough to the true value, that is, all operating points should be located on the diagonal line, the distance between each operating point and the diagonal line means the prediction error of the point, and the prediction error of a good model should be as small as possible. The prediction results show that, compared with GPR with the square exponential kernel function, the GPR model with the rational quadratic kernel function could achieve a higher *R*^2^ value (*R*^2^ = 0.99) and lower RMSE value (7.9321), MSE value (62.919), and MAE value (3.2494). However, the GPR with the combined kernel function has a *R*^2^ value of 1.00, the RMSE value is reduced to 3.262, and the MSE value and MAE value of the combined kernel function are also lowered.

**Table 2:** GPR Training Result with Different Kernel Functions^a^

	RMSE	*R*^2^	MSE	MAE
GPR with the squared exponential kernel	16.287	0.97	265.27	5.8231
GPR with the rational quadratic kernel	7.9321	0.99	62.919	3.2494
GPR with the combined kernel	1.8060	1.00	3.262	1.2807

a GPU is used for parallel computing.

## 3 Construction of Engine Black Box Model

**Figure 4:**
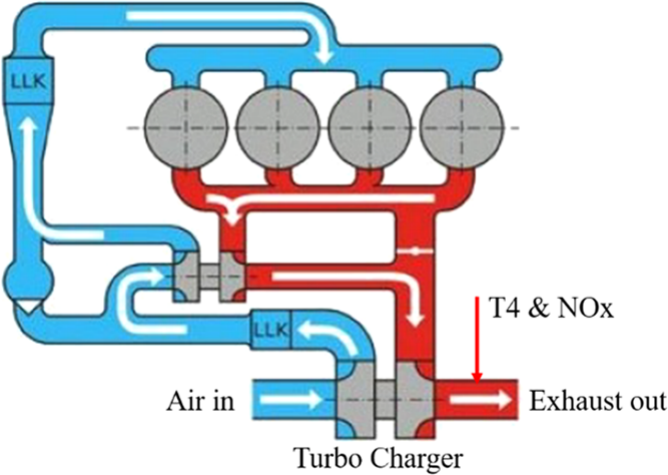
Engine schematic.

**Figure 5:**
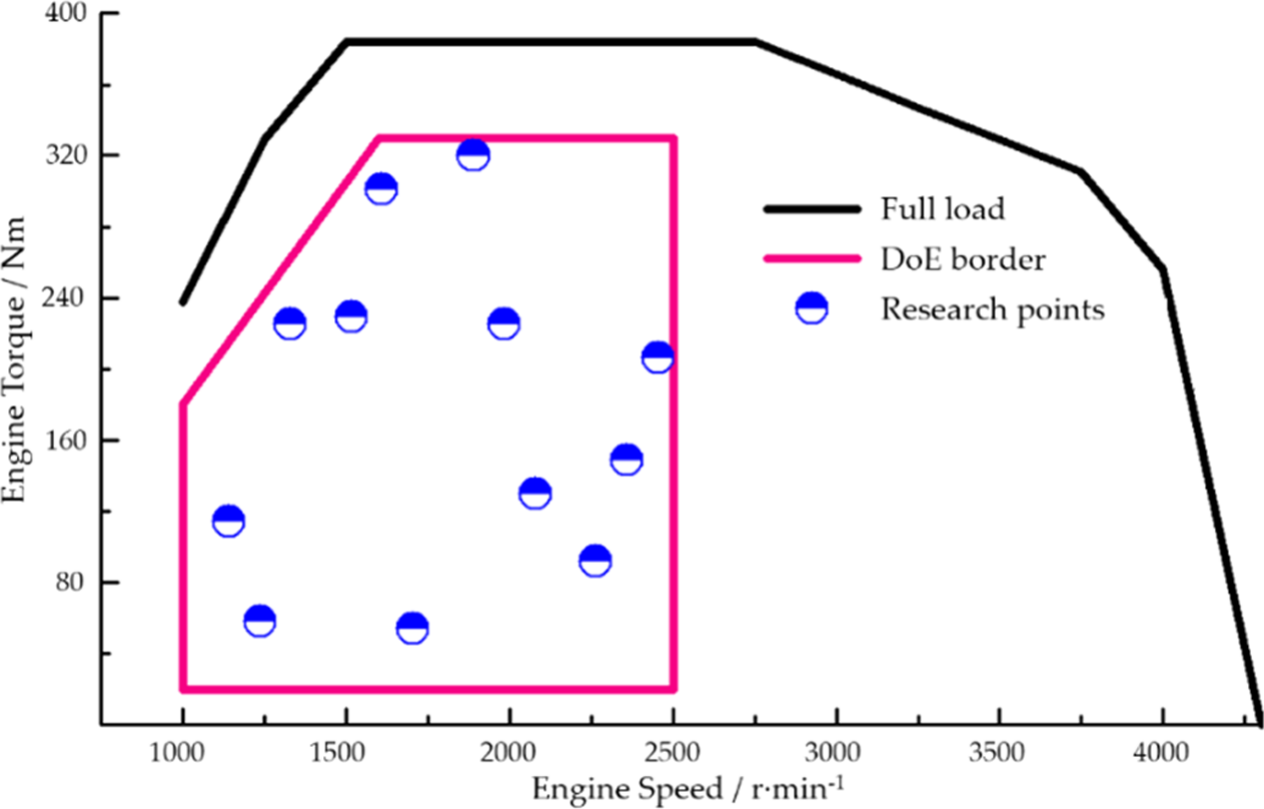
Research points covered by the dataset.

Engine operating conditions change rapidly and are influenced by many factors. As is shown in Figure 4, the operating data of the engine under steady-state DoE test conditions are taken as sample data^25^ for the construction of an engine black box model. The main influencing factors of engine torque, exhaust gas temperature after turbo (shown as T4 in Figure 4), and NOx raw emission (shown as NOx in Figure 4) are taken into consideration. The research points covered by the dataset are shown in Figure 5.

**Figure 6:**
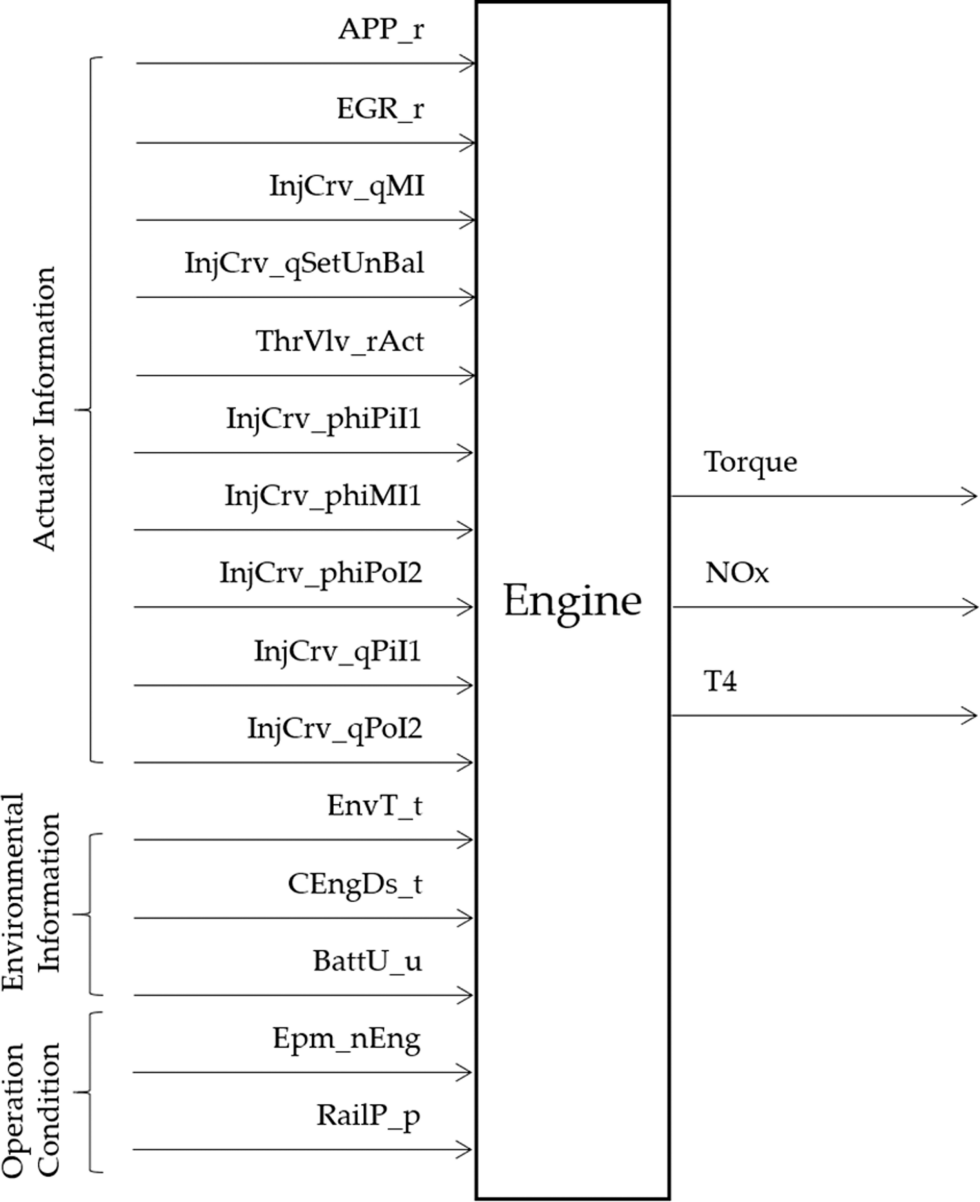
Schematic diagram of the engine black box system.

As shown in Figure 6, if the engine is regarded as a black box system, the input information can be divided into the following three categories with a total of 15 input signals:

1 Actuator information, 10 signals—APP_r (accelerator pedal percentage), EGR_r (exhaust gas recirculation valve percentage), InjCrv_qMI (main injection quantity), InjCrv_qSetUnBal (total injection quantity), ThrVlv_rAct (throttle valve percentage), InjCrv_phiPiI1 (pilot injection 1 angle), InjCrv_phiMI1 (main injection angle), InjCrv_phiPoI2 (postinjection 2 angle), InjCrv_qPiI1 (pilot injection 1 quantity), and InjCrv_qPoI2 (postinjection 2 quantity);
2 Engine operating environment information, three signals—EnvT_t (ambient temperature), CEngDs_t (engine coolant temperature), and BattU_u (engine battery voltage);
3 Engine running status information, two signals—Epm_nEng (engine speed) and RailP_p (rail pressure of common rail fuel injection system).

## 4 GPR-Based Engine Model Training

In this study, the operating data under the DoE test condition is used as the training dataset, and the combined kernel function is used for the fitting of the engine black box system. Version information of the main tools used is shown in Table 3. The computer used is a mobile workstation equipped with an 8-core/16-thread processor and an NVIDIA Quadro T600 discrete graphics card, and GPU parallel computing is used to accelerate the training process.

**Table 3:** Tool Information

tools	version information
MATLAB	version 9.10 (R2021a)
deep learning toolbox	version 14.2
statistics and machine learning toolbox	version 12.1
parallel computing toolbox	version 7.4

### 4.1 Training of Engine Torque Model

The GPR-based model training process is mainly composed of two parts: hyperparameter optimization and data fitting.

As shown in eq 9, the kernel function of the regression model used in this study is weighted by the square exponential kernel function and rational quadratic kernel function. After further sorting, the combined kernel function can be expressed as eq 15.
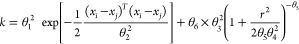
15where θ_1_ is the standard deviation of the signal in the square exponential kernel function, θ_2_ is the scale of the signal feature length in the square exponential kernel function, θ_3_ is the standard deviation of the signal in the rational quadratic kernel function, θ_4_ is the length of the signal feature in the rational quadratic kernel function, θ_5_ is the scale mixing parameter of the rational quadratic kernel function, and θ_6_ is the weight coefficient of the rational quadratic kernel function in the combined kernel function.

**Figure 7:**
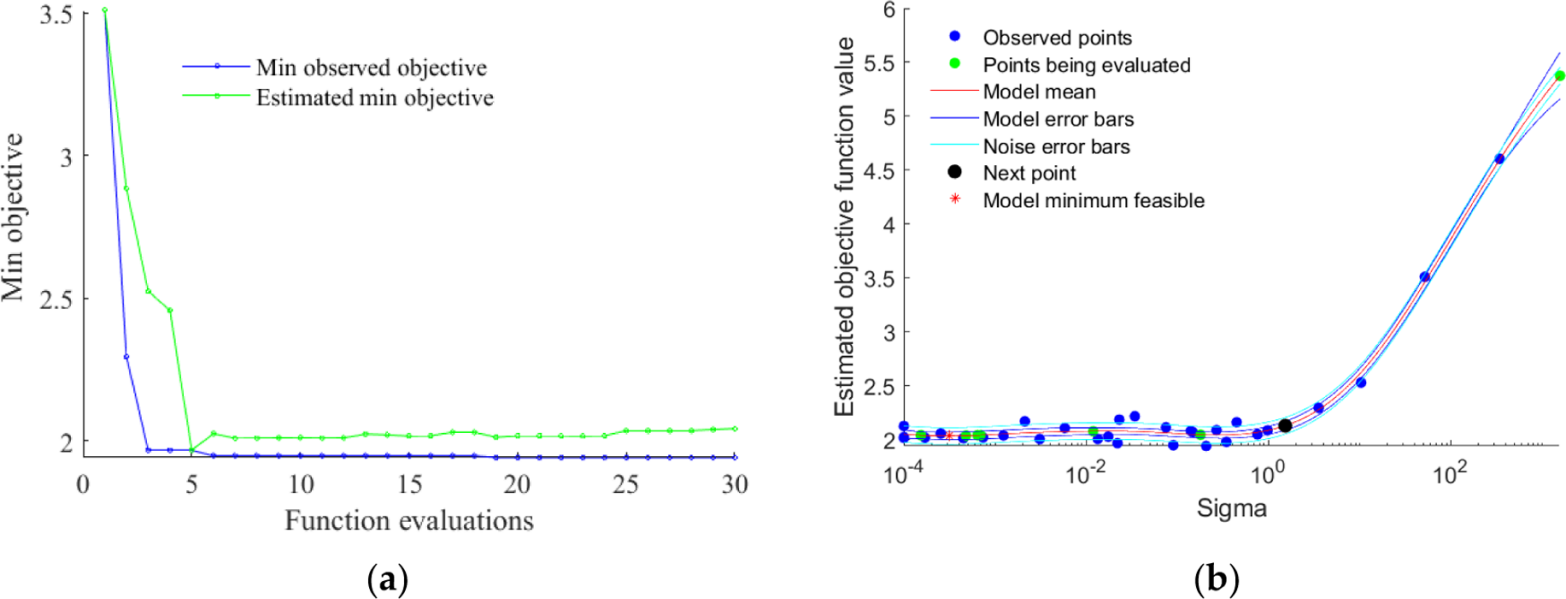
Hyperparameter value fitting plot: (a) variation of the minimum objective value with function evaluations; (b) variation of the estimated objective function value with different sigma values.

It can be seen from eq 15 that there are six hyperparameters: θ_1_–θ_6_. The optimization process of hyperparameters is the process of finding the optimal solution of θ_1_–θ_6_. The algorithm is designed to find hyperparameters that minimize fivefold cross-validation loss by using automatic hyperparameter optimization. As shown in Table 4 and Figure 7, after 30 iterations, the observed best objective function value is 1.6747, and the standard deviation of the dataset (shown as sigma in the table) is 0.00010001. The obtained hyperparameter optimal solution is shown in Table 5.

**Table 4:** Iterative Fitting Process for Hyperparameters

iteration	active workers	eval result	objective: log(1 + loss)	objective runtime	bestsofar (observed)	bestsofar (estim.)	sigma
1	8	best	2.8198	1499.2	2.8198	2.8198	23.245
2	8	best	1.6928	1930.3	1.6928	1.7527	0.00011607
3	8	best	1.6916	2003.1	1.6916	1.7458	0.10798
4	8	accept	1.8157	2040.9	1.6916	1.6916	0.25442
5	8	accept	1.7151	2042.7	1.6916	1.692	0.031193
6	8	accept	1.7796	2043.9	1.6916	1.6983	0.88557
7	8	accept	1.819	1979.5	1.6916	1.6918	0.00021741
8	8	accept	1.7869	1916.2	1.6916	1.7242	0.0005629
9	8	accept	1.7659	2042.8	1.6916	1.735	0.074124
10	8	accept	1.7739	2045.1	1.6916	1.7339	0.0022856
11	8	accept	1.7694	2050.4	1.6916	1.7339	0.00010002
12	8	accept	6.0762	5409.5	1.6916	1.737	819.24
13	8	accept	5.7821	6033.3	1.6916	1.7361	708.63
14	8	accept	1.7222	2189.1	1.6916	1.7262	0.011508
15	8	accept	4.8688	4670.2	1.6916	1.7226	372.45
16	8	accept	1.7966	2220.4	1.6916	1.7452	0.039516
17	8	accept	1.7491	2127.8	1.6916	1.7457	0.016879
18	8	best	1.6747	2207.9	1.6747	1.7161	0.00010001
19	8	accept	1.7589	2202.8	1.6747	1.7154	0.024322
20	8	accept	1.9357	1973.7	1.6747	1.7148	3.3463
21	8	accept	1.69	2189.4	1.6747	1.7141	0.005429
22	8	accept	1.72	2133.9	1.6747	1.7151	0.0011314
23	8	accept	1.705	2133.6	1.6747	1.7148	1.5404
24	8	accept	1.7	2211.5	1.6747	1.7165	0.0099237
25	8	accept	1.7516	2173.2	1.6747	1.7164	0.00010032
26	8	accept	1.7519	2248.1	1.6747	1.7161	0.00010015
27	8	accept	1.7126	2172.2	1.6747	1.7159	0.13824
28	8	accept	2.3887	1972.9	1.6747	1.7138	8.3652
29	8	accept	3.6048	1881.1	1.6747	1.7184	74.358
30	8	accept	1.7374	2040.4	1.6747	1.7182	0.47048

**Table 5:** Hyperparameter Values Obtained after Training

hyperparameters	value
θ_1_	0.8663
θ_2_	0.6700
θ_3_	4.9035
θ_4_	2.2162
θ_5_	1.3625
θ_6_	2.1214

In this study, the norm value from functional analysis theory is used to measure the discrete degree of dataset in the vector space. The L2 norm value, also known as Euclidean norm, is defined as the distance between all elements in the vector and the origin point, the calculation formula is shown in eq 16; the infinity norm is defined as the absolute value of the largest element in the vector, and its calculation formula is shown in eq 17. The L2 norm and infinite norm characterize the degree of dispersion between sample data and fitting results.
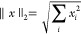
16
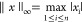
17

**Figure 8:**
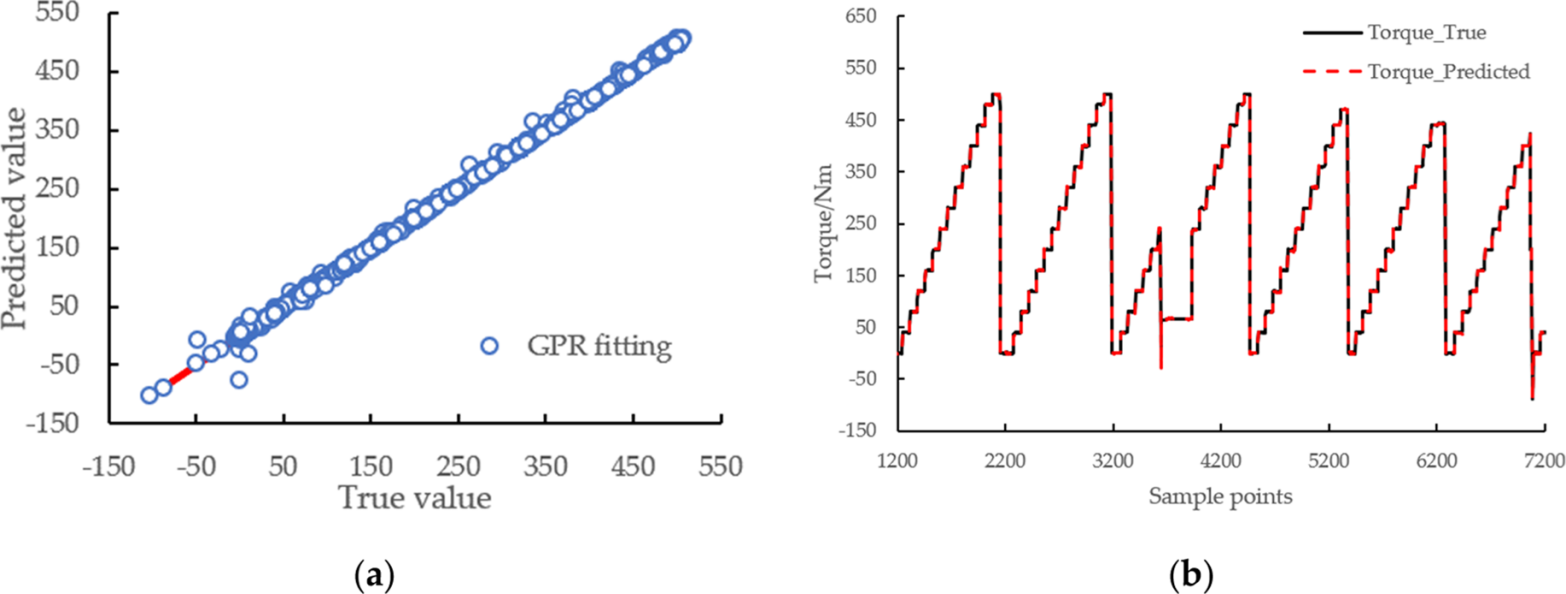
Engine torque fitting result with GPR: (a) deviation plot of predicted and actual values of engine torque; (b) engine torque fitting results (only a subset of data sample points is shown).

The fitting results are shown in Table 6, Figure 8 and Table 7. The results show that the infinite norm of the final gradient is 37.96 (shown as norm grad in the table), the L2 norm at the final step is 0.1074 (shown as the norm step in the table), the relative infinite norm of the final gradient is 0.008030, the degree of dispersion between the predicted value and the actual value of engine torque is small, *R*^2^ reaches 1.00, RMSE is 1.7381, MSE is 3.0211, and MAE is 1.0077.

**Table 6:** Fitting Process for Hyperparameters

iteration	fun value	norm grad	norm step	curv	gamma	alpha	accept
0	4.06 × 10^4^	5.15 × 10^4^	0.00 × 10^0^		1.94 × 10^–5^	0.00 × 10^0^	yes
1	9.33 × 10^3^	7.59 × 10^3^	1.19 × 10^0^	ok	2.23 × 10^–5^	1.00 × 10^0^	yes
2	8.02 × 10^3^	5.57 × 10^3^	1.86 × 10^–1^	ok	8.29 × 10^–5^	1.00 × 10^0^	yes
3	6.00 × 10^3^	2.30 × 10^3^	5.10 × 10^–1^	ok	1.41 × 10^–4^	1.00 × 10^0^	yes
4	5.38 × 10^3^	1.13 × 10^3^	3.59 × 10^–1^	ok	2.80 × 10^–4^	1.00 × 10^0^	yes
5	5.08 × 10^3^	4.84 × 10^2^	3.71 × 10^–1^	ok	5.02 × 10^–4^	1.00 × 10^0^	yes
6	4.91 × 10^3^	2.41 × 10^2^	4.08 × 10^–1^	ok	1.39 × 10^–3^	1.00 × 10^0^	yes
7	4.80 × 10^3^	1.46 × 10^2^	5.36 × 10^–1^	ok	2.85 × 10^–3^	1.00 × 10^0^	yes
8	4.75 × 10^3^	6.98 × 10^1^	5.79 × 10^–1^	ok	2.75 × 10^–3^	1.00 × 10^0^	yes
9	4.73 × 10^3^	1.01 × 10^2^	4.79 × 10^–1^	ok	1.43 × 10^–3^	1.00 × 10^0^	yes
10	4.73 × 10^3^	3.80 × 10^1^	1.07 × 10^–1^	ok	6.62 × 10^–4^	1.00 × 10^0^	yes

**Table 7:** Engine Torque Model Training Result

item	value
*R*^2^	1.00
RMSE	1.7381
MSE	3.0211
MAE	1.0077

### 4.2 Comparison with Other Commonly Used Fitting Methods for Engine Torque Prediction

In recent years, with the continuous in-depth exploration of machine learning technology, researchers have proposed and verified many prediction techniques, such as linear regression, decision tree, SVM, GPR, neural network, and so forth. These prediction methods have a wide range of applications in the field of deep learning. However, for engine performance prediction, the performance of different prediction methods varies widely.

The same training dataset used in this study is used for prediction comparison of engine torque performance using different data fitting methods included in the officially released Regression Learner APP from MathWorks; the fitting result is shown in Table 8.

**Table 8:** Engine Torque Prediction with Different Fitting Methods

			fitting result (validation^a^)			
	fitting methods	hyperparameters	RMSE	*R*^2^	MSE	MAE
	GPR introduced in this study	combined kernel defined in this study	1.7381	1.00	3.0211	1.0077
linear regression	linear	preset: linear robust option: off	11.34	1.00	128.59	8.1743
	interaction linear	preset: interactions linear robust option: off	7.7276	1.00	59.715	4.2302
	robust linear	preset: robust linear robust option: on	11.786	0.99	138.91	7.9663
decision tree	fine tree	minimum leaf size: 4 surrogate decision splits: off	6.1395	1.00	37.694	1.6206
	medium tree	minimum leaf size: 12 surrogate decision splits: off	6.0582	1.00	36.702	1.672
	coarse tree	minimum leaf size: 36 surrogate decision splits: off	6.6141	1.00	43.747	1.8936
	boosted trees	minimum leaf size: 8preset: boosted trees	14.932	0.99	222.96	11.591
	bagged tree	minimum leaf size: 8preset: bagged trees	5.149	1.00	26.512	1.341
SVM	linear SVM	kernel function: linear kernel scale: automatic	12.409	0.99	153.99	9.7683
	quadratic SVM	kernel function: quadratic kernel scale: automatic	10.492	1.00	110.08	8.0499
	fine Gaussian SVM	kernel function: Gaussian kernel scale: 0.97	12.908	0.99	166.62	9.952
	medium Gaussian SVM	kernel function: Gaussian kernel scale: 3.9	10.949	1.00	119.88	8.7303
	coarse Gaussian SVM	kernel function: Gaussian kernel scale: 15	10.394	1.00	108.03	7.6224
neural network	narrow neural network	number of fully connected layers: 1; first layer size: 10;activation: ReLu	7.1358	1.00	50.92	3.8654
	medium neural network	number of fully connected layers: 1; first layer size: 25;activation: ReLu	6.1125	1.00	37.362	2.8717
	bilayered neural network	number of fully connected layers: 2; first layer size: 10; second layer size: 10;activation: ReLu	6.69	1.00	44.756	3.326
	trilayered neural network	number of fully connected layers: 3; first layer size: 10; second layer size: 10; third layer size: 10;activation: ReLu	12.728	0.99	162	6.4575

a Validation data are 5% randomly selected from the training dataset.

Comparison results show that

1. For linear regression fitting methods, compared to linear (RMSE = 11.34) and robust linear regression (RMSE = 11.786), interaction linear can achieve a lower RMSE value (RMSE = 7.7276) because interaction linear regression adds interaction terms to the regression model, and this is helpful to explore relationships between variables;
2. Bagged tree achieves the lowest RMSE value (RMSE = 5.149), except for the method proposed in this study. Unlike other decision tree algorithms, bagged tree uses many trees for data fitting, and this could help to leverage the insight of many models;
3. SVM is a linear classifier that performs binary classification of data in a supervised learning manner. SVM performs well in classification problems but performs poorly in engine torque prediction.
4. The neural network has the characteristics of large-scale parallel processing, distributed storage, elastic topology, high redundancy, and nonlinear operation. The medium neural network achieves a relatively lower RMSE value (RMSE = 6.1125) in torque prediction.
5. The GPR algorithm based on the combined kernel function proposed in this study has the lowest RMSE value (RMSE = 1.7381).

### 4.3 Training of T4 and NOx Emission Models

**Figure 9:**
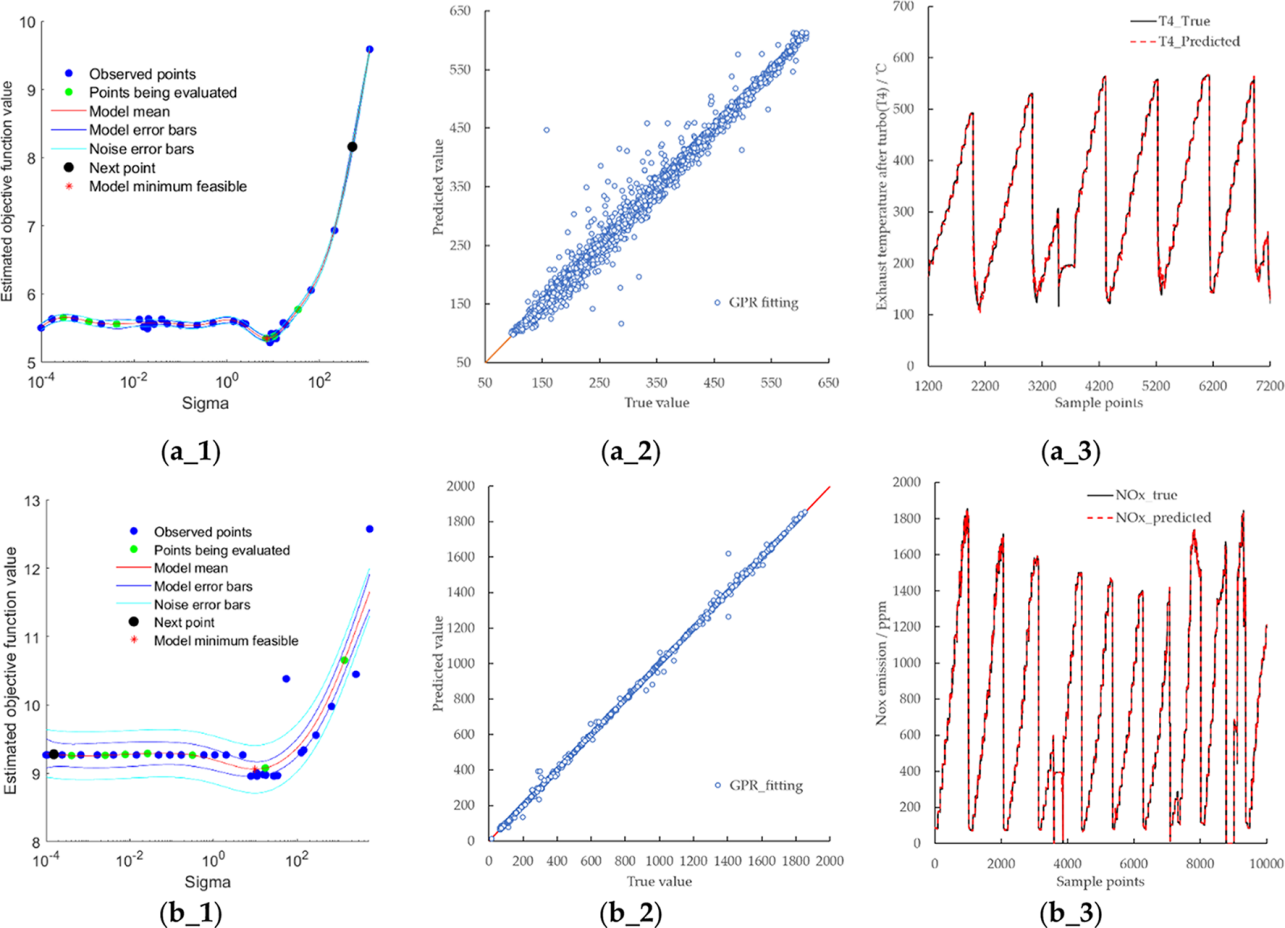
Fitting results of T4, NOx, and soot: (a_1) change of T4 objective function value with sigma; (a_2) deviation plot of predicted and actual values of T4; (a_3) comparison of predicted and actual T4 values; (b_1) change of the NOx objective function value with sigma; (b_2) deviation plot of predicted and actual values of NOx raw emissions from engine; (b_3) comparison of predicted and actual NOx values.

In this study, data modeling of T4 and NOx emissions is carried out. The modeling results are shown in Figure 9 and Tables 9 and 10.

**Table 9:** Hyperparameter Values Obtained after Training

hyperparameters	value_T4^a^	value_NOx^b^
θ_1_	1.6085	5.2944
θ_2_	0.6986	–2.4635
θ_3_	4.3196	4.9019
θ_4_	0.6815	–1.5238
θ_5_	0.9611	0.0521
θ_6_	2.0553	2.8000

a Corresponding values for T4 prediction.

b Corresponding values for NOx prediction.

**Table 10:** Model Training Result

item	value_T4	value_NOx
Inf norm grad final^a^	81.05	95.53
two norm step final^b^	0.3889	5.844 × 10^–3^
Inf norm grad final^c^	9.488 × 10^–3^	8.092 × 10^–3^
*R*^2^	1.0000	0.9999
RMSE	10.5446	9.1829
MSE	111.1887	84.3262
MAE	5.0621	2.1530

a Infinity norm of the final gradient.

b L2 norm of the final step.

c Relative infinity norm of the final gradient.

The fitting results of T4 and NOx emissions show that the infinite norm of the final gradient is 81.05 and 95.53, the L2 norm of the final step is 0.3889 and 5.844 × 10^–3^, and the relative infinite norm of the final gradient is 9.488 × 10^–3^ and 8.092 × 10^–3^ with *R*^2^ of 1.0000 and 0.9999. The results show that the accuracy of the model trained by the GPR fitting method based on the combined kernel function is high.

### 4.4 Verification of the Generalization Ability of the Model

To verify the generalization prediction accuracy of the constructed engine model, the actual operating data of the same type of engine under the World Harmonized Transient Cycle (WHTC) condition is used in this study as the validation dataset, 1817 samples are included in this dataset with a sample rate of 1 s. This validation dataset is a brand new dataset that the model has never seen during the training process.

**Figure 10:**
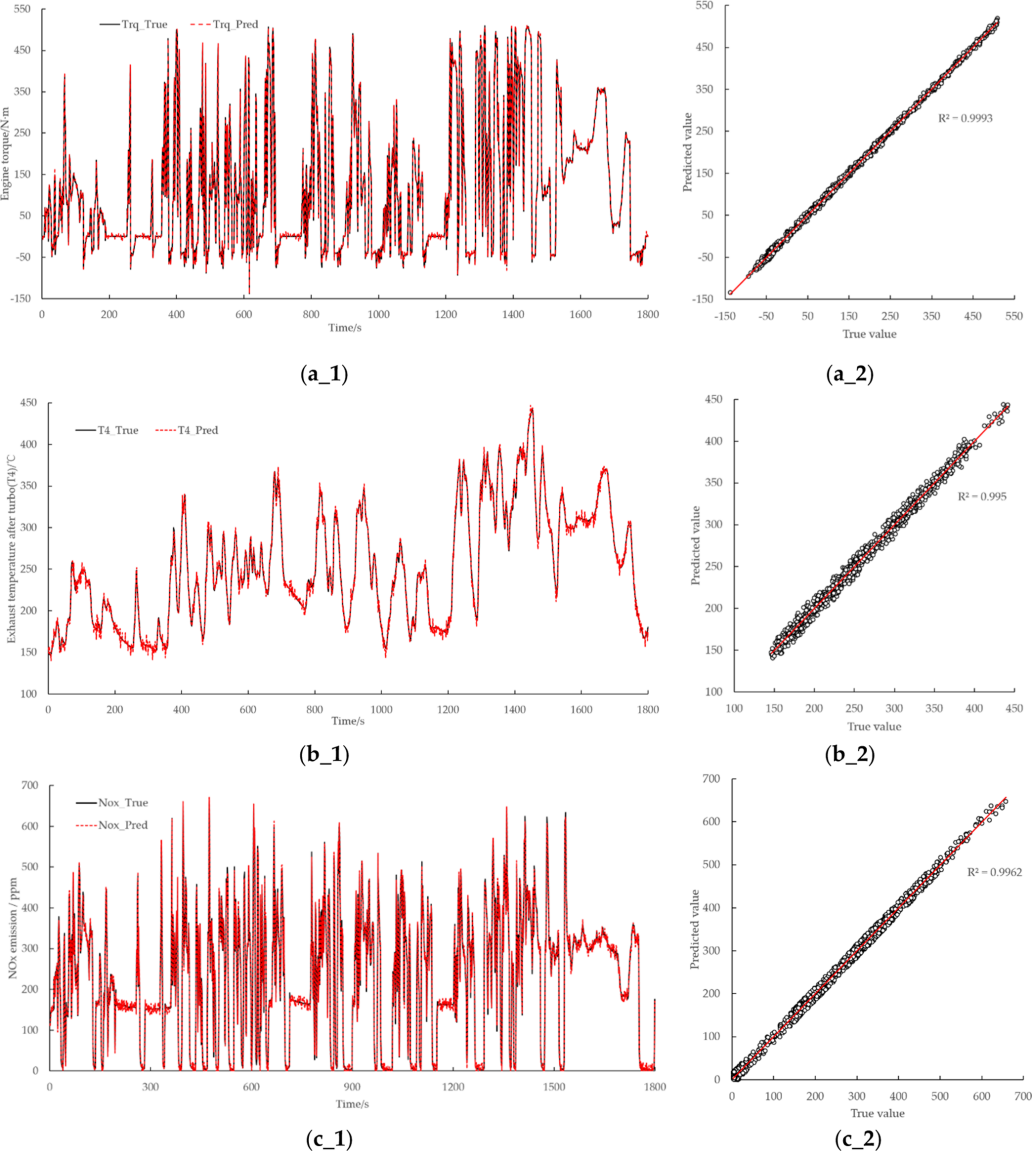
Validation results of the GPR model based on the combined kernel function under WHTC: (a_1) validation results of engine torque under WHTC; (a_2) *R*^2^ result of engine torque under WHTC; (b_1) validation results of T4 under WHTC; (b_2) *R*^2^ result of T4 under WHTC; (c_1) validation results of NOx emission under WHTC; (c_2) *R*^2^ result of NOx emission in WHTC.

The model verification results are shown in Figure 10. Under transient conditions, the errors of engine torque, T4, and NOx emission results are small. The *R*^2^ value of engine torque prediction result is 0.9993, the *R*^2^ value of T4 prediction is 0.995, and the *R*^2^ value of NOx emission prediction is 0.9962. The results show that GPR technique based on the combined kernel function adopted in this study could be applied for engine performance prediction (shown as torque prediction in this study), temperature prediction (shown as T4 temperature prediction in this study), and emission prediction (shown as NOx prediction in this study).

## 5 Conclusions

In this study, we explore the application of GPR technology based on a combined kernel function in the fields of engine torque prediction, temperature prediction, and emission prediction. The above analyses lead to the following conclusions:

1. Compared with the square exponential kernel function and rational quadratic kernel function, the combined kernel function constructed in this study could not only have the advantage of square exponential kernel function in modeling with high-dimensional samples but also improve the dynamic response performance through the rational quadratic kernel function;
2. The comparison results with linear regression, decision tree, SVM, neural network, and Gaussian regression show that GPR technique based on the combined kernel function proposed in this study could achieve higher prediction accuracy in the fields of engine torque prediction, emission prediction (NOx emission prediction), and exhaust temperature prediction (T4 temperature prediction). The *R*^2^ values of engine torque prediction and T4 prediction reach 1.00, and the *R*^2^ value of NOx prediction model reaches 0.9999;
3. The generalization ability verification results of the prediction model show that for the new data the model has not seen during the training process, the *R*^2^ value of engine torque calculation result is 0.9993, the *R*^2^ value of T4 is 0.995, and the *R*^2^ value of NOx emission result is 0.9962, results show that for the data not included in the training dataset, the model can still achieve high prediction accuracy;
4. The Gaussian regression technique based on the combined kernel function proposed in this study is suitable for both engine prediction under steady-state operating conditions (as shown by the model training results) and engine prediction under transient conditions (as shown in the model’s generalized verification test).

As mentioned above, the GPR algorithm based on combined kernel function proposed in this study can effectively improve engine performance simulation accuracy, and further research can be carried out in the fields of engine/vehicle virtual calibration, DoE design, and hardware-in-the-loop real-time simulation.

## Abbreviations

NOx: nitrogen oxides
EGR: exhaust gas recirculation
RMSE: root-mean-square error
*R*^2^: goodness of fit
MSE: mean square error
MAE: mean absolute error
